# Amylin and Calcitonin: Potential Therapeutic Strategies to Reduce Body Weight and Liver Fat

**DOI:** 10.3389/fendo.2020.617400

**Published:** 2021-01-08

**Authors:** David S. Mathiesen, Asger Lund, Tina Vilsbøll, Filip K. Knop, Jonatan I. Bagger

**Affiliations:** ^1^ Center for Clinical Metabolic Research, Gentofte Hospital, Hellerup, Denmark; ^2^ Steno Diabetes Center Copenhagen, Gentofte, Denmark; ^3^ Department of Clinical Medicine, Faculty of Health and Medical Sciences, University of Copenhagen, Copenhagen, Denmark; ^4^ Novo Nordisk Foundation Center for Basic Metabolic Research, Faculty of Health and Medical Sciences, University of Copenhagen, Copenhagen, Denmark

**Keywords:** amylin, calcitonin, dual amylin-calcitonin receptor agonist, DACRA, NAFLD, non-alcoholic fatty liver disease, obesity, pramlintide

## Abstract

The hormones amylin and calcitonin interact with receptors within the same family to exert their effects on the human organism. Calcitonin, derived from thyroid C cells, is known for its inhibitory effect on osteoclasts. Calcitonin of mammalian origin promotes insulin sensitivity, while the more potent calcitonin extracted from salmon additionally inhibits gastric emptying, promotes gallbladder relaxation, increases energy expenditure and induces satiety as well as weight loss. Amylin, derived from pancreatic beta cells, regulates plasma glucose by delaying gastric emptying after meal ingestion, and modulates glucagon secretion and central satiety signals in the brain. Thus, both hormones seem to have metabolic effects of relevance in the context of non-alcoholic fatty liver disease (NAFLD) and other metabolic diseases. In rats, studies with dual amylin and calcitonin receptor agonists have demonstrated robust body weight loss, improved glucose tolerance and a decreased deposition of fat in liver tissue beyond what is observed after a body weight loss. The translational aspects of these preclinical data currently remain unknown. Here, we describe the physiology, pathophysiology, and pharmacological effects of amylin and calcitonin and review preclinical and clinical findings alluding to the future potential of amylin and calcitonin-based drugs for the treatment of obesity and NAFLD.

## Introduction

Hepatic steatosis is widely regarded the hepatic manifestation of the metabolic syndrome (1). In parallel with the increasing prevalence of obesity and its related diseases, non-alcoholic fatty liver disease (NAFLD) is currently the most widespread liver disease in the world (2–4). Body weight loss is currently the most effective strategy to improve both measures of steatosis and NAFLD outcomes (5) and several anti-obesity medications in clinical development have demonstrated improvements with regards to liver fat content (6, 7). Recent preclinical studies have demonstrated body weight loss, reduced hepatic steatosis and metabolic improvements in rats following administration of novel dual amylin and calcitonin receptor agonists (DACRAs) (8–13).

NAFLD is defined by increased liver fat content (>5%) without significant alcohol consumption or steatosis caused by any other mechanism (e.g., medications, hepatitis, autoimmunity or inheritable diseases) (14, 15). NAFLD covers a spectrum of stages, ranging from simple accumulation of liver fat to non-alcoholic steatohepatitis (NASH) with inflammation and ultimately to hepatic fibrosis and cirrhosis (1). Recently, the term “metabolic-associated fatty liver disease (MASH)” has been proposed as a unifying definition of hepatic steatosis in individuals with overweight/obesity, metabolic dysregulation and/or manifest type 2 diabetes (16). This definition recognizes the importance of obesity and insulin resistance, rather than the absence of excessive alcohol consumption, as a causal factor for the development of hepatic steatosis. Resonating with this, the prevalence of NAFLD increases dramatically with the number of metabolic syndrome criteria present in a population (17). Furthermore, the well-established association between NAFLD and type 2 diabetes highlights a potential casual relationship between impaired glucose metabolism with insulin resistance and hepatic steatosis (18). Even in prediabetes, increased liver fat content is a central characteristic (19). Hepatic lipid accumulation may facilitate resistance to both insulin (20) and glucagon (21), which are important pathophysiological characteristics of type 2 diabetes (22, 23). Insulin resistance is regarded a driver of hepatic steatosis through increased hepatic lipogenesis and exaggerated tissue lipolysis, ultimately increasing accumulation of fatty acids in the liver (24).

Currently, bariatric surgery is the most effective weight loss therapy, but it is costly, associates with a non-negligible risk of complications and not all patients are eligible for surgery (25). Therefore, pharmacotherapies to reduce body weight are being vigorously pursued, and amylin as well as DACRAs are emerging as potential novel anti-obesity drug candidates, especially in combination with other body weight-lowering gastrointestinal peptides.

This review summarises amylin and calcitonin physiology and pathophysiology in obesity and NAFLD and provides insight into the potential therapeutic role of pharmacological doses of amylin and calcitonin of relevance to metabolic diseases including obesity and NAFLD.

## Amylin

**Figure 1:**
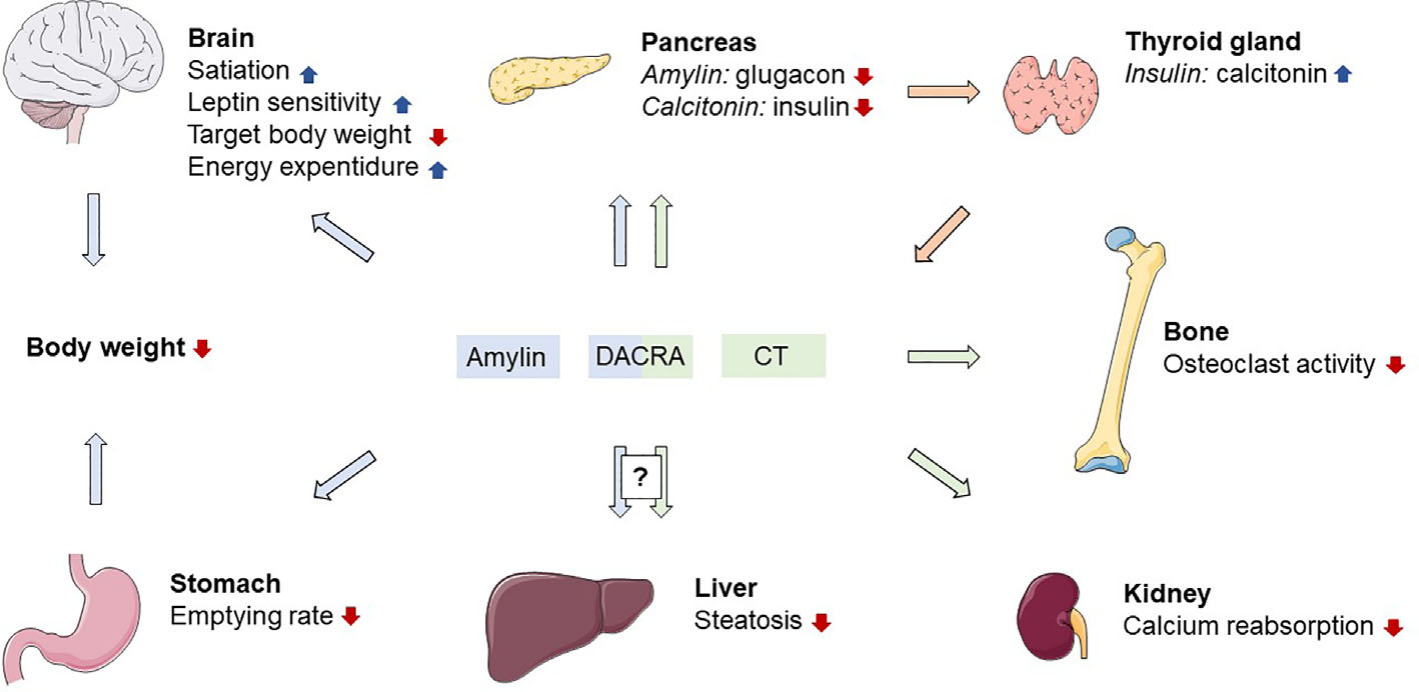
Proposed physiological actions of amylin and calcitonin receptor activation. DACRA, dual amylin and calcitonin receptor agonist; CT, calcitonin; blue arrows indicates effects related to amylin; green arrows indicates effects related to calcitonin; orange arrows indicates effects related to insulin. Figure elements from smart.servier.com under CC BY 3.0.

Amylin is a 37-amino acid peptide hormone (**Table 1**) mainly produced in the pancreatic beta cells and co-secreted with insulin in response to ingested nutrients (**Figure 1**) (28, 29). The hormone has a well-established role as a satiety signal; an effect that is mediated *via* direct action on amylin receptors in specific areas of the brain, i.e., area postrema and the nucleus of the solitary tract (30, 31). Amylin is also an efficacious inhibitor of gastric emptying, which further facilitates satiation (32–34), and may suppress glucagon secretion *via* central mechanisms (35, 36). There are several isotypes of the amylin receptor (37) which all are G protein-coupled receptors consisting of two units; a core unit constituted by the calcitonin receptor (7-transmembrane receptor) and one of three receptor activity-modifying proteins (RAMP1-3) (37). Stimulating the amylin receptor complex increases the production of intracellular cyclic adenosine monophosphate (38, 39). The tissue distribution of the amylin receptor complex is difficult to describe for a number of reasons: 1) the calcitonin receptor (the core unit) has two subtypes which interact with RAMPs, 2) RAMPs are associated with other receptors than the amylin receptor complex, and 3) there is a lack of selective pharmacological tools and antibodies to target specific amylin (RAMP/calcitonin) receptor complexes (26). Current data indicate that several brain regions including the area postrema and hypothalamus are important sites for amylin action (40, 41). In a knock-out mouse model, lack of the calcitonin receptor specifically in proopiomelanocortin-expressing neurons in the hypothalamus lead to increased adiposity, glucose intolerance and decreased energy expenditure (41). Furthermore, amylin receptors are not selectively activated by amylin alone and interact indiscriminately with other hormones of similar structure (i.e., calcitonin, calcitonin gene-related peptide, and adrenomedullin) (42). Calcitonin, for instance, has been shown to activate several subtypes of the amylin receptor (42). In a similar manner, it has been shown that amylin has affinity for both calcitonin and amylin receptors (43). Several amylin receptor antagonists have been identified, but as alluded to above, these compounds also have selectivity issues, and do not distinguish between amylin receptor subtypes (26). Therefore, the importance of the individual receptor in mediating the endogenous actions of amylin is difficult to establish.

**Table 1:** Amino acid sequences of amylin, calcitonin and related peptides.

		1							7																														37	
rAMY		K	C		N	T	A	T	C	A	T	Q	R	L	A	N	F	L	V	R	S	S	N	N	L	G	P	V	L	P	P	T	N	V	G	S	N	T	Y	-NH_2_
hAMY		K	C		N	T	A	T	C	A	T	Q	R	L	A	N	F	L	V	H	S	S	N	N	F	G	A	I	L	S	S	T	N	V	G	S	N	T	Y	-NH_2_
Pramlintide		K	C		N	T	A	T	C	A	T	Q	R	L	A	N	F	L	V	H	S	S	N	N	F	G	P	I	L	P	P	T	N	V	G	S	N	T	Y	-NH_2_
Davalintide		K	C		N	T	A	T	C	V	L	G	R	L	S	Q	E	L	H	R					L		Q	T	Y	P	R	T	N	T	G	S	N	T	Y	-NH_2_
sCT			C	S	N	L	S	T	C	V	L	G	K	L	S	Q	E	L	H	K					L		Q	T	Y	P	R	T	N	T	G	S	G	T	P	-NH_2_
hCT			C	G	N	L	S	T	C	M	L	G	T	Y	T	Q	>D	F	N	K					F		H	T	F	P	Q	T	A	I	G	V	G	A	P	-NH_2_
eCT			C	S	N	L	S	T	C	V	L	G	K	L	S	Q	E	L	H	K					L		Q	T	Y	P	R	T	D	V	G	A	G	T	P	-NH_2_
KBP-042	Ac-		C	S	N	L	S	T	C	V	L	G	K	L	S	Q	E	L	H	K					L		Q	T	Y	P	R	T	D	V	G	A	N	A	P	-NH_2_
KBP-089	Ac-		C	S	N	L	S	T	C	M	L	G	R	L	S	Q	S	L	H	R					L		Q	T	Y	P	K	T	D	V	G	A	N	A	P	-NH_2_

The C-terminus is amidated (-NH_2_) in all peptides, whereas only novel dual amylin and calcitonin receptor agonist (DACRA) compounds have an N-terminus acetylation (Ac-) for enhanced stability. rAMY, rat amylin; hAMY, human amylin; sCT, salmon calcitonin; hCT, human calcitonin; eCT, eel calcitonin, blue indicates amino acid sequence derived from human amylin; green indicates amino acid sequence derived from salmon calcitonin; yellow indicates amino acids derived from rat amylin; gray indicates amino acid sequence derived from human calcitonin; red indicates amino acids derived from eel calcitonin. Adapted from (8, 26, 27).

## The Role of Amylin in Obesity

Several studies point to a role of amylin in the hormonal regulation of food intake and body weight. Amylin has several characteristics of a satiating hormone: 1) it is released after food ingestion (28, 29), 2) it has a short half-life (~13 min) with rapid onset of action (44, 45), and 3) it dose-dependently decreases food intake when administered to rats mainly in supraphysiological doses (40, 45, 46). This effect also translates into human trials with amylin receptor agonism (see further details below). The role of endogenous amylin as a satiating agent is supported by the observation that injection of the amylin receptor antagonist AC187 intravenously or directly into the area postrema acutely increases food intake in rats (47, 48). In addition to its effect on satiety, preclinical studies suggest that endogenous amylin also has the characteristics of an adiposity signal (i.e., a body weight-regulatory hormonal factor circulating in proportion with body fat mass), much like insulin and leptin, namely the ability to increase energy expenditure and lower body weight *via* central mechanisms (49, 50). In rats, chronic intravenous administration of amylin leads to body weight loss and diminished fat deposition, whereas centrally administered amylin, in addition to lowering body weight, also seems to reduce the target body weight set by the brain (49–52). Supporting its role as an endogenous adiposity signal, acute and chronic amylin antagonism with AC187 has the opposing effect and increases food intake and body weight of rats (50). As an adiposity signal, amylin enhances the satiating effect of cholecystokinin (CCK). This is evidenced by the synergistic acute effect of co-administered CCK and amylin on food intake when infused intraperitoneally in mice (53) and further supported by the diminished action of CCK in mice models of amylin deficiency (54) and in rats after infusion of amylin receptor antagonists (55, 56). Chronic intraperitoneal infusion of CCK decreases food intake in rats, but has little effect on body weight due to a compensatory increase in meal frequency (57). Contrary to this, chronic subcutaneous infusion of amylin reduces both meal size and frequency with concomitant body weight loss in rats (46). Interestingly, amylin also interacts with leptin in the control of energy metabolism in a number of preclinical studies, supporting its combined role as a satiety and adiposity signal (58–61). In rats with obesity or functional leptin resistance, the effect of amylin agonism on eating behaviour is still observed for 24 h after injection of an amylin agonist (58). Furthermore, intraventricular administration of leptin seems to enhance the inhibitory effect of amylin on short-term eating, suggesting synergism in the actions of these hormones (59). Additionally, amylin re-sensitizes obese rats to leptin when co-administered with a leptin agonist for 14 days (60). As opposed to leptin, amylin show a preserved anorectic response when investigating obese and hyperamylinaemic rats (61). In fact, several rodent studies suggest a well-preserved anorectic response to acute amylin administration in otherwise obese and leptin-resistant states (26, 60, 61), thus making amylin a promising candidate for pharmaceutical weight loss therapy. Furthermore, an additive effect on weight loss is also observed in obese individuals when amylin and leptin agonists are chronically co-administered, adding further evidence to the interaction between leptin and amylin (60). The chemical properties of human amylin predisposes the hormone to aggregate and form amyloid fibrils, which are often found in pancreatic islets of individuals with type 2 diabetes and possibly contribute to beta cell destruction (62). For this reason, infusions of human amylin are difficult to perform, often requiring highly supraphysiological dosages to elicit little or no effect (63, 64). However, several stable amylin analogues with the ability to induce body weight loss have been developed (further details below).

Taken together, amylin has acute effects as a satiety signal combined with homeostatic effects of an adiposity signal on body weight, suggesting an important role of amylin in the regulation of body mass. In clinical trials, circulating amylin levels seem to tightly correlate with fat mass. Studies indicate that basal and glucose or meal-stimulated levels of amylin are elevated in individuals with obesity (65–74). This may relate to the role of amylin as a regulator of body mass but could also be a manifestation of the increased beta cell secretory activity often found in obesity. These studies are generally limited by their sample size and contrasting reports have been published (75). Preclinical studies support the notion of elevated amylin levels in rats with obesity (76, 77). This might be a result of decreased amylin sensitivity following prolonged hyperamylinaemia, but there is currently no evidence of this in humans. More studies designed to specifically evaluate amylin secretion and sensitivity in individuals with obesity compared to individuals with normal weight are needed.

## Calcitonin

Calcitonin is a 32-amino acid peptide hormone (**Table 1**) derived from the 116-amino acid precursor pro-calcitonin and secreted from the C cells of the thyroid gland. As alluded to above, calcitonin mediates its effects *via* the 7-transmembrane calcitonin receptor and a subsequent increase in intracellular cAMP (38). Due to the interaction with RAMPs, the tissue distribution of the monomeric calcitonin receptor is challenging to elaborate, but well-known target organs of calcitonin include bones and kidneys (78, 79). In humans the secretion of calcitonin is stimulated by ingestion of calcium (80). Calcitonin has a strong hypocalcaemic effect *via* inhibition of osteoclasts (**Figure 1**) (79) and promotion of renal excretion of calcium, presumably by inhibiting tubular reabsorption of calcium (79). Since the discovery of calcitonin in 1962 (81), great effort has been put into the description of its inhibitory effect on osteoclasts and the increased calcium excretion in humans whereas any other physiological effects have not been described. Human calcitonin is often considered a rudimentary hormone, mainly due to the fact that hypersecretion or deficiency of calcitonin (as seen in patients with thyroid medullary cancer) is not associated with bone abnormalities (82). Furthermore, the more potent form of calcitonin originating from salmon has been the preferred choice of treatment for chronic conditions with hypercalcaemia until better antiresorptive drugs emerged (e.g., bisphosphonates, denosumab and raloxifen) (83).

## Calcitonin in Metabolic Disease

It is difficult to evaluate the role of endogenous calcitonin in metabolic diseases for a number of reasons: 1) only few studies have applied human calcitonin in humans, 2) there are currently no antagonists available which selectively target the monomeric calcitonin receptor (26), and 3) studies applying the more potent salmon calcitonin reveal effects attributable to amylin receptor activity as well (84). As reviewed in the following sections, the actions of salmon and human calcitonin are not directly comparable. A few studies have used mammalian calcitonin to investigate effects beyond those related to calcium and bone metabolism. Interestingly, both human and porcine calcitonin infusions inhibit the insulin response to acute glucose administration in humans (85–87). But whether this effect has physiological relevance remains to be determined. In a study with 26 subjects, who were mainly overweight but with normal glucose tolerance, there was an increase in serum calcitonin levels after a 75-g oral glucose tolerance test which correlated with insulin levels, suggesting a possible relationship between insulin and calcitonin (88). This is in concert with the observation that insulin directly stimulates calcitonin release in the perfused pig thyroid gland (89). Additionally, higher endogenous calcitonin levels in individuals with obesity has been reported (90). Finally, procalcitonin is expressed in adipose tissue and its expression associates with obesity, insulin resistance and metabolic syndrome (91). Taken together, research thus far gives some indication that a correlation between calcitonin and insulin might exist in man, but studies designed specifically to affirm this are warranted.

## Amylin and Calcitonin-Based Pharmacotherapies

### Pramlintide

Pramlintide is a Food and Drug Administration (FDA)-approved amylin analogue, developed for individuals with type 1 diabetes or insulin-treated type 2 diabetes as an adjunct therapy to mealtime insulin (92). Pramlintide has pharmacological properties comparable to human amylin, but with enhanced stability, thus making it suitable for subcutaneous administration in humans (44). It is a relatively short-lived peptide with a half-life of ~20–45 min in humans, thus requiring administration with every meal to diminish postprandial plasma glucose excursions (44, 93). Inspired by rat amylin, which is less prone to dimerize, the enhanced stability of pramlintide compared to human amylin was achieved by introducing three amino acid substitutions (pro25,28,29) into the sequence of human amylin (26) (**Table 1**). In addition to reducing postprandial plasma glucose excursions, pramlintide has demonstrated body weight-lowering capabilities in several clinical trials (94–112). In a 6-week randomised, blinded, placebo-controlled multicentre trial, 60 obese individuals (average body mass index (BMI) = 35.3 kg/m^2^) were titrated to 180 µg pramlintide injected subcutaneously before each meal to test the translatability of the body weight loss observed in trials with pramlintide in individuals with diabetes (94). After 6 weeks, the mean change in body weight from baseline was -2.04 kg, corresponding to a weight loss of roughly 2%. This was highly significant compared to the placebo group. In a 4-month trial of similar design, the majority (88%) of 108 obese individuals (average BMI = 37.9 kg/m^2^) were titrated to 240 µg meal time pramlintide injected subcutaneously three times daily (95). The resulting average body weight loss of 3.7% was highly significant compared to the placebo group. To evaluate the sustainability of these results, a 12-month randomised, double-blinded, placebo-controlled multicentre trial with 146 obese subjects (average BMI ~ 37 kg/m^2^) evaluated the effect of pramlintide combined with life-style intervention on long-term body weight loss (96). An initial 4-month dose escalation period evaluated different doses (120, 240, and 360 µg) and administration frequencies (two or three times daily) for pramlintide and was followed by an 8-month extension of the pre-assigned pramlintide treatment regime. At 4 months, body weight reduction was comparable to previous pramlintide trials, ranging from 3.8 to 6.1 kg depending on pramlintide dose. Interestingly, after 12 months, the body weight loss was sustained across all pramlintide treatment regimens except for those treated with 120 µg twice daily. This is encouraging, as gradual body weight regain is a common observation following lifestyle-induced weight loss (97, 113, 114). In obese individuals with insulin-treated type 2 diabetes, pramlintide dose-dependently reduces body weight (98–104). The body weight-lowering effect of pramlintide is also seen in individuals with type 1 diabetes (105–112).

As outlined above, pramlintide treatment is associated with 2%–6% reductions in body weight across several patient categories, including individuals with obesity and type 2 diabetes, who are prone to develop hepatic steatosis. This makes amylin-based pharmacology a promising candidate for the treatment of obesity and NAFLD. However, long-term pramlintide treatment is limited by low bioavailability and the short half-life of the drug, which makes it less suitable for chronic therapy due to the high administration frequency (two to three times daily) and therefore high compliance-related demands. Additionally, pramlintide monotherapy only has a modest effect on body weight loss compared to for example glucagon-like peptide 1 (GLP-1) receptor agonists (115) or the more dramatic changes observed after bariatric surgery (116). Pharmacotherapies targeting several mechanisms are currently being extensively explored as potential weight loss strategies (117). In preclinical and clinical settings, amylin has been combined with several other compounds with weight regulatory abilities to elicit beneficial effects on body weight. These include agents based on peptide YY (PYY), CCK, melanocortins, leptin, and GLP-1 as well as small molecule anorectics (phentermine/sibutramine) (54, 118–123). Only the GLP-1/amylin combination is currently being developed for obesity treatment in clinical trials (124). Later in this section, we review the effect of targeting multiple receptors in the calcitonin receptor family *via* novel unimolecular dual agonists, but first we briefly consider results from studies with other amylin-based agents.

### Davalintide

Davalintide is a 32-amino acid peptide amylin receptor agonist (**Table 1**) with enhanced potency, efficacy and duration of action compared to amylin in rats (125). Davalintide was developed in response to the low bioavailability and short half-life which rendered pramlintide therapy for obesity inefficient. The peptide is a chimera of amylin and salmon calcitonin and shares 49% of the amino acid sequence of rat amylin and pramlintide (26, 125). The half-life of davalintide is 26 min, and thus, comparable to rat amylin (126). However, davalintide reduces food consumption in rats for up to 23 h, compared to only 6 h with rat amylin (125). Further, davalintide dose-dependently reduces body weight and fat mass in rats with approximately 2-fold greater efficacy than rat amylin (125). The prolonged duration is likely explained by slow receptor disassociation of the salmon calcitonin portion of davalintide (38, 127). Indeed, receptor binding analysis revealed very limited receptor disassociation of davalintide in the rat nucleus accumbens (126). As of now, these preclinical studies with davalintide represent the only available literature on davalintide and further development of davalintide in humans trials have been discontinued due to lack of superiority to pramlintide on weight loss (128). Nevertheless, these few trials with davalintide illustrate how the calcitonin receptor system may constitute a target for the treatment of obesity and associated metabolic conditions such as NAFLD.

### Long-Acting Amylin Agonists

Amylin has been modified by various methods (e.g., by adding a polyethylene glycol (PEG), glycosylation, or albumin binding motif to the molecule) to extend its half-life and reduce the frequency of administration, thus making it more suitable for chronic use in body weight loss therapy (129–135). In mice, subcutaneous administration of PEGylated amylin acutely reduces glycaemia with prolonged action compared to unmodified amylin (129). In rat models of type 1 diabetes, a PEGylated amylin analogue prevented meal-induced hyperglycaemia and promoted sustained normoglycaemia up to 8 h after injection of the amylin analogue (132). Importantly, acute and chronic studies show that long-acting amylin analogues decrease body weight and energy intake in rats (136, 137). Given the convenience of a once-daily injection compared to several daily injections, long-acting amylin agonists are attractive to develop and are currently being pursued as novel anti-obesity and anti-diabetes drug candidates by multiple pharmaceutical companies. Novo Nordisk is currently testing a long-acting amylin analogue, AM833, in overweight and obese individuals in phase I and II clinical trials (138, 139). In a recently published phase II trial, the effect of life style interventions along with increasing doses (0.3, 0.6, 1.2, 2.4 and 4.5 mg) of AM833 once weekly on body weight was investigated 706 individuals with obesity/overweight (140). At 26 weeks, body weight had decreased progressively and dose-dependently without plateau, with reductions ranging from 6%–10.8%. Compared to placebo and 3 mg liraglutide once daily, the observed weight loss amongst participants receiving AM833 was significantly greater for all doses of AM833 versus placebo and for 4.5 mg once weekly versus liraglutide. AM833 has also been evaluated for use in combination with the GLP-1 analogue semaglutide in a phase I clinical trial (124, 141). A total of 80 participants with obesity or overweight were treated with ascending doses of AM833 in combination with 2.4 mg semaglutide once-weekly (141). After 20 weeks, the participants receiving the highest dose of AM833 with semaglutide lost an average of 17.1% body weight from baseline (141). Also, Zealand Pharma is developing long-acting amylin analogues for treatment of obesity and diabetes (142, 143). Preclinical data from diabetic and obese rats models have been released for the compounds ZP4982 and ZP5461; both molecules are potent activators of calcitonin and amylin receptors and effectively lower blood glucose and body weight (142, 143). Compared to twice-daily preclinical dosing with the GLP-1 analogue liraglutide, once-weekly dosing of ZP4982 at almost equimolar doses was superior in terms of body weight loss in an obese rat model (143). A phase I clinical trial was conducted with a long-acting amylin analogue developed by Zealand Pharma in cooperation with Boehringer Ingelheim in 2018, but the collaboration on this analogue was terminated in 2020 (144, 145).

### Salmon Calcitonin

Calcitonin extracted from salmon displays prolonged receptor activation and binding in humans compared with human calcitonin (38). It is also superior to mammalian calcitonin with regards to its hypocalcaemic effects in rats and humans (146). Interestingly, salmon calcitonin has effects beyond those related to bone metabolism. In human studies, salmon calcitonin inhibits gastric emptying and gastrin release following a meal while evoking a dose-dependent relaxation of the gallbladder both in the postprandial and fasting state (147, 148). In mice and monkeys, salmon calcitonin acts anorectically and causes weight loss after a single administration (149, 150). In chronic studies, oral preparations of salmon calcitonin also reduce food intake and body weight in rat models of obesity and diabetes (151, 152). Furthermore, salmon calcitonin acutely stimulates energy expenditure during food restriction in rats (153). Human and salmon calcitonin only have a 50% amino acid sequence homology (38) (**Table 1**), and rodent studies applying amylin receptor antagonists suggest that the anorectic effect of salmon calcitonin results at least partially from amylin receptor activation (84). *In vitro*, salmon calcitonin displays superior binding affinity at amylin receptors with no discrimination between amylin and calcitonin receptors (154). Compared to human calcitonin, salmon calcitonin also displays prolonged activation of human calcitonin receptors when tested in mammalian cell lines expressing the calcitonin receptor (38). This suggests that salmon calcitonin is a dual agonist with potency at amylin *and* calcitonin receptors, highlighting the possibility of targeting these receptors using a single molecule with dual-receptor agonistic properties.

### Dual Amylin and Calcitonin Receptor Agonists

Inspired by the pharmacology of salmon calcitonin, DACRAs for the treatment of obesity and diabetes have been developed (8–13). DACRAs display equal affinity and enhanced potency at amylin and calcitonin receptors compared to salmon calcitonin (8). In Zucker diabetic fatty rats, 4-week treatment with the DACRA KBP-042 from Nordic Bioscience was compared with salmon calcitonin and vehicle in a pair-fed design; showing significant weight loss and improved glucose tolerance compared to both vehicle and salmon calcitonin (8). In other studies, subcutaneous injections with KBP-042 lead to substantial body weight loss with alleviation of leptin and insulin resistance in rats on high-fat diet compared to rats on normal diet (11, 12). Interestingly, after a 7-week treatment period, a reduction in liver fat deposition was observed in the KBP-042-treated rats, but not in a pair-fed control group of rats (12). The ability to reduce hepatic lipid accumulation has also been demonstrated with another DACRA, KBP-089 (10). In rats subjected to a high-fat diet without therapy for 10 weeks and subsequently to subcutaneous peptide therapy for 8 weeks, KBP-089 completely abolished the hepatic steatosis achieved by the initial high-fat feeding (10). Importantly, this effect was not observed in a pair-fed group of rats, suggesting that DACRA therapy has beneficial effects on hepatic steatosis beyond those related to reduced food intake and weight loss. These peptides show promise in terms of their effects on body weight and several physiological parameters related to energy homeostasis and glucose metabolism in rodent models of obesity and diabetes. From the available literature, it is not clear to which degree the amylin and calcitonin receptor mediates the beneficial results obtained in preclinical DACRA studies. In a rodent study comparing the activity of a DACRA molecule to native amylin, calcitonin and the combination amylin/calcitonin, calcitonin receptor activation did not appear to be important for the weight lowering and satiating abilities of the DACRA molecule, which were primarily mediated by the amylin receptor (155). On the other hand, calcitonin and amylin had additive effects on fasting glycaemia, suggesting that calcitonin receptor activity may facilitate some of the metabolic improvements of DACRA molecules after all (155).

A 12-week phase II placebo-controlled clinical trial with 255 patients with type 2 diabetes receiving KBP-042 has been completed (156), but further development of KBP-042 has been terminated by Eli Lilly and Nordic Bioscience to focus on the development of KBP-089, which is reportedly superior to KBP-042 (157, 158). KBP-089 is currently being tested in a phase I clinical trial in patients with type 2 diabetes (159). As of now, no results from clinical trials with DACRA molecules have been published. Thus, it remains to be established whether the promising preclinical results obtained with DACRA peptides will translate into possible treatment strategies for metabolic diseases such as obesity and NAFLD.

## Conclusions

A substantial amount of literature describes beneficial metabolic effects of compounds activating amylin and calcitonin receptors separately or in combination. These effects include body weight loss and reduced hepatic lipid accumulation, which are important cornerstones in the treatment of obesity and NAFLD. Pharmaceutical companies are pursuing strategies based on amylin and calcitonin as viable alternatives to bariatric surgery, the currently most effective treatment option for obesity.

Preclinical and clinical data support amylin as an anti-obesity hormone, whereas the role of calcitonin in obesity remains more uncertain. Nevertheless, salmon calcitonin, like new compounds such as long-acting amylin analogues and DACRAs, demonstrates a potential for combined amylin and calcitonin receptor agonism as a future treatment strategy for obesity and related conditions such as NAFLD. Disentangling the effects of these dual agonists through specific amylin and calcitonin pathways may prove to be difficult without specific receptor antagonists. Nevertheless, it is relevant to evaluate from a clinical perspective in order to optimize the effects of pharmacotherapy targeting amylin and calcitonin receptors. Data from the clinical programs investigating the new amylin and DACRA compounds will be very interesting to follow since long-acting agonists with greater selectivity at amylin or calcitonin receptors may help elucidate this. However, dedicated studies are needed to test the translatability of the preclinical data of amylin and calcitonin dual agonism and to delineate the separate and combined physiological effects of amylin and/or calcitonin receptor activity in humans.

## Author Contributions

DM drafted the manuscript. AL, TV, FK, and JB reviewed and edited the manuscript. All authors contributed to the article and approved the submitted version.

## Conflict of Interest

The authors declare that the research was conducted in the absence of any commercial or financial relationships that could be construed as a potential conflict of interest.
